# Therapeutic strategies focusing on immune dysregulation and neuroinflammation in rosacea

**DOI:** 10.3389/fimmu.2024.1403798

**Published:** 2024-07-29

**Authors:** Kuan-Yi Tu, Chiau-Jing Jung, Yi-Hsien Shih, Anne Lynn S. Chang

**Affiliations:** ^1^ Division of General Medicine, Taipei Medical University Shuang Ho Hospital, New Taipei, Taiwan; ^2^ Graduate Institute of Medical Sciences, College of Medicine, Taipei Medical University, Taipei, Taiwan; ^3^ Department of Microbiology and Immunology, School of Medicine, College of Medicine, Taipei Medical University, Taipei, Taiwan; ^4^ Department of Dermatology, School of Medicine, College of Medicine, Taipei Medical University, Taipei, Taiwan; ^5^ Department of Dermatology, Taipei Medical University Shuang Ho Hospital, New Taipei, Taiwan; ^6^ Department of Dermatology, Stanford University School of Medicine, Redwood City, CA, United States

**Keywords:** rosacea, immune dysregulation, neuroinflammation, microbiota, therapeutics

## Abstract

Rosacea is a complex inflammatory condition characterized by papulopustular lesions and erythema on the central face for which there is no cure. The development of rosacea is influenced by both external triggers and genetics, but the common pathophysiology is overactivation of the immune system. Here, we review the current data on proinflammatory cytokines and dysregulation of the neurovascular system as targetable components of rosacea. Amelioration of cutaneous and gastrointestinal dysbiosis and other external factors impacts the immune state and has been observed to improve rosacea. While multiple treatments exist, many patients do not achieve their goals for rosacea control and highlights an unmet need for dermatologic care. Current interventions encompass topical/oral drugs, light devices, and avoidance of triggers management. Additional understanding of the underlying pathogenesis may help us develop novel targeted therapeutic strategies to improve rosacea.

## 1 Introduction

Rosacea is a chronic, relapsing, inflammatory skin disease characterized by symptoms such as burning, stinging, erythema, and papulopustular lesions on the central face (1). To date, rosacea is considered a multifactorial disease, but our current understand has not led to a cure, nor sufficient control of the disease in many cases. Both immune dysregulation and neuroinflammation have been implicated in the development of rosacea. Here, we review the current literature on these components (2–4) which may lead to novel targets and pathways for future clinical trials.

For instance, rosacea can worsen due to various external trigger factors of the immune system, such as ultraviolet (UV) radiation and *Demodex* mites (5, 6). Neuroinflammation is also significant in rosacea, triggered by factors like alcohol, spicy foods, and temperature changes, with activation of calcium channels and vasodilation manifest as skin redness, a common symptom of the condition (3).

## 2 Pathophysiology

**Figure 1:**
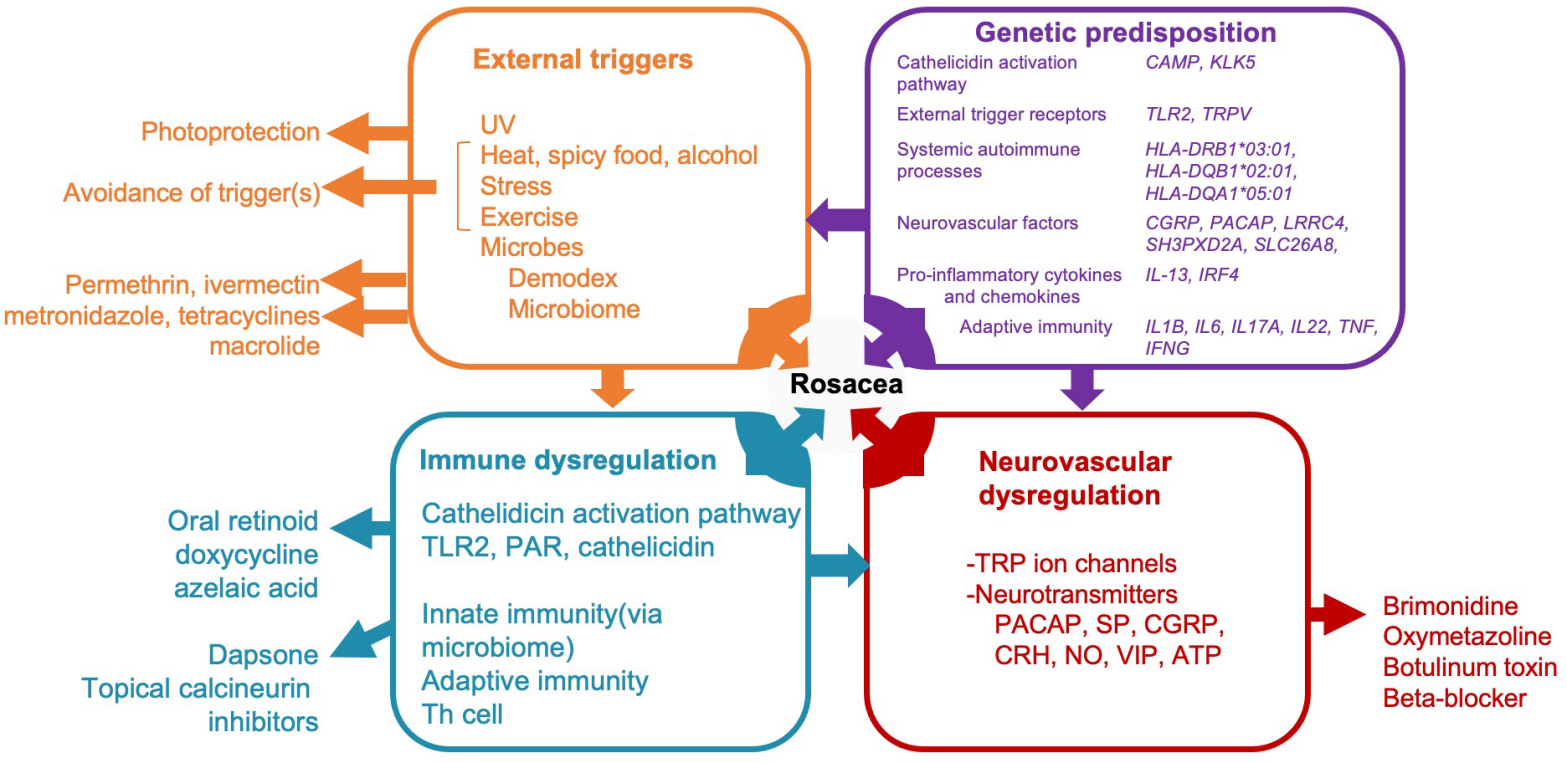
A diagram illustrating potential pathogenic mechanisms of rosacea categorized into four major categories and interaction with immune system. Rosacea, a multifactorial condition, involves external triggers (orange box), genetic predisposition (purple box), immune dysregulation (blue box), and neurovascular dysregulation (red box). Treatment modalities correspond to these mechanisms, as shown outside of the boxes in the diagram. Only drugs aligned with Swiss S1 and National Rosacea Society guidelines are indicated. UV, ultraviolet; CAMP, cathelicidin antimicrobial peptide; KLK-5, Kallikrein 5; TLR2, toll-like receptor 2; TRP-, transient receptor potential channel; CGRP, calcitonin gene related peptide; PACAP, pituitary adenylate cyclase-activating peptide; IL-, interleukin; IRF4, interferon regulatory factor-4; TNF, tumor necrosis factor; IFNG, Interferon gamma; PAR, protease-activated receptor; SP, substance P; CRH, corticotropin-releasing hormone; NO, Nitric oxide; VIP, vasoactive intestinal polypeptide; ATP, adenosine triphosphate.

The precise pathophysiology of rosacea continues to be investigated. So far, rosacea is known to be a multifactorial, chronic, inflammatory skin disease (7, 8). The contributing pathogenic mechanisms of rosacea can be categorized into four major categories which can interact with each other: external triggers (e.g., exposure to UV radiation and *Demodex* mites), genetic predisposition, immune dysregulation, and neurovascular dysregulation (2) (**Figure 1**).

Rosacea may be exacerbated by external factors such as ultraviolet (UV) radiation, alcohol, spicy food, extreme temperatures, and *Demodex* mites and other microbes (1). Exposure to UV radiation and *Demodex* mites activates Toll-like receptor 2 (TLR2), leading to the activation and subsequent release of cathelicidin—an antimicrobial peptide integral to the pathogenesis of rosacea (5, 6). Kallikrein 5 (KLK5) converts cathelicidin into its active form, LL-37, which has been implicated in inflammation, angiogenesis, and telangiectasis in rosacea (9).

Neuroinflammation is a major component of rosacea (4), though it is unclear if the initial trigger is immunologic, leading to stimulation of nerves that mediate itch and burning or vice versa. Some data exists that the nerves can be activated first by common triggers leading to blood vessel dilation. For instance, triggers such as alcohol and spicy food consumption and temperature changes can activate calcium channels, specifically transient receptor potential vanilloid (TRPV)1/transient receptor potential ankyrin (TRPA)1, thereby inducing the release of neurotransmitters and the dilatation of microvessels (3). These changes manifest as skin erythema, a characteristic clinical sign of rosacea.

The following sections shed more light on these mechanisms of inflammation and the components of the immune system involved in rosacea (**Figure 1**).

### 2.1 External triggers of inflammation and the immune system

Exposure to UV radiation and external pathogens activates TLR2 (5, 10), leading to the subsequent activation and release of KLK5 (11). In addition, inflammatory cells such as neutrophils and macrophages release matrix metalloproteinases (MMPs), which convert pro-KLK5 into KLK5 (12). KLK5 is a serine protease that cleaves cathelicidin, an antimicrobial peptide secreted by epidermal keratinocytes, into the human antimicrobial peptide LL-37, an immune-stimulating factor (9). This process, referred to as the cathelicidin activation pathway, plays a crucial role in the inflammatory response in rosacea (9). Given that LL-37 plays a pivotal role in rosacea-related inflammation, this pathway is frequently referenced in the subsequent discussion on immune dysregulation.

Regarding the triggers of rosacea, researchers have extensively focused on the cutaneous microbiome (13). Rosacea may be associated with certain skin microorganisms such as *Demodex folliculorum*, *Staphylococcus epidermidis*, *Bacillus oleronius* (a bacterium carried by *Demodex* mites), *Bartonella quintana*, and *Chlamydia pneumoniae* (8). However, studies examining the relationship between these microorganisms and rosacea have reported inconsistent results. Among these microorganisms, *Demodex* has garnered the most attention and has the largest evidence for its role in rosacea. *Demodex*, with its chitin exoskeleton, can increase TLR2 levels and induce a proinflammatory response (5). An *in vitro* study revealed that *Demodex* antigens can activate the human inflammasome (5). Furthermore, *Demodex*-associated microorganisms, such as *B. oleronius*, may induce the differentiation of immune cells and promote the secretion of various substances, including cathelicidin, MMP-9, tumor necrosis factor (TNF), and interleukin (IL)-8 (14). The current literature strongly supports the role of *Demodex* as a trigger of rosacea.

### 2.2 Genetic predisposition including immune associated genes

Recent epidemiological studies, family and twin studies, genetic association studies, and *in vitro* analyses have increasingly suggested the involvement of a genetic component in the development of rosacea (6, 15–20). By including both identical and fraternal twins, a twin study differentiated genetic susceptibility from the role of environmental factors in rosacea development; the results suggested that genetics contribute to 46% of individuals’ predisposition to rosacea (19).

The genes mentioned in the literature can be broadly classified into the following major categories:

1. Cathelicidin activation pathway: Overexpression of genes such as cathelicidin antimicrobial peptides (6), *KLK5*, and *LL-37* (21) has been validated in molecular and functional studies using human keratinocytes and skin explants of rosacea compared to healthy skin controls.
2. External triggers: RNA sequencing revealed alteration of gene expression levels such as *TLR2* and *TRPV* in samples from rosacea patients compared to non-lesional skins or healthy skin controls (22–25).
3. Systemic autoimmune processes: A genome wide association study (GWAS) of Caucasian rosacea patients without any autoimmune disease found single nucleotide polymorphism (SNP) in genes such as HLA-DRB1*03:01, HLA-DQB1*02:01, and HLA-DQA1*05:01, which are associated with MHC class II and antigen presentation (26).
4. Neurovascular factors: The roles of neurotransmitters such as calcitonin gene-related peptide (*CGRP*) and pituitary adenylate cyclase-activating peptide (*PACAP*) in rosacea were supported by a case-control observational study and a retrospective study (27–29). Moreover, a whole-genome sequencing in 3 large rosacea families and whole exome sequencing in 49 additional validation families from Han population which revealed rare, single deleterious variants of *LRRC4*, *SH3PXD2A*, and *SLC26A8*, which are genes for neural synaptic processes and cell adhesion, in large families with rosacea. Subsequent *in vitro* and mouse studies have revealed that these genes induced the production of vasoactive neuropeptides, thereby leading to rosacea-like skin inflammation (4).
5. Proinflammatory cytokines and chemokines: Another GWAS study of Caucasian rosacea patients found SNP in genes such as interleukin-13 (*IL-13*) and interferon regulatory factor-4 (*IRF4*) (30).
6. Altered adaptive immunity: Genes related to the Th1/Th17 pathway, specifically IL1B, IL6, IL17A, and IL22 are upregulated in rosacea patients compared to healthy skin controls (23, 24, 31).

### 2.3 Immune dysregulation

**Figure 2:**
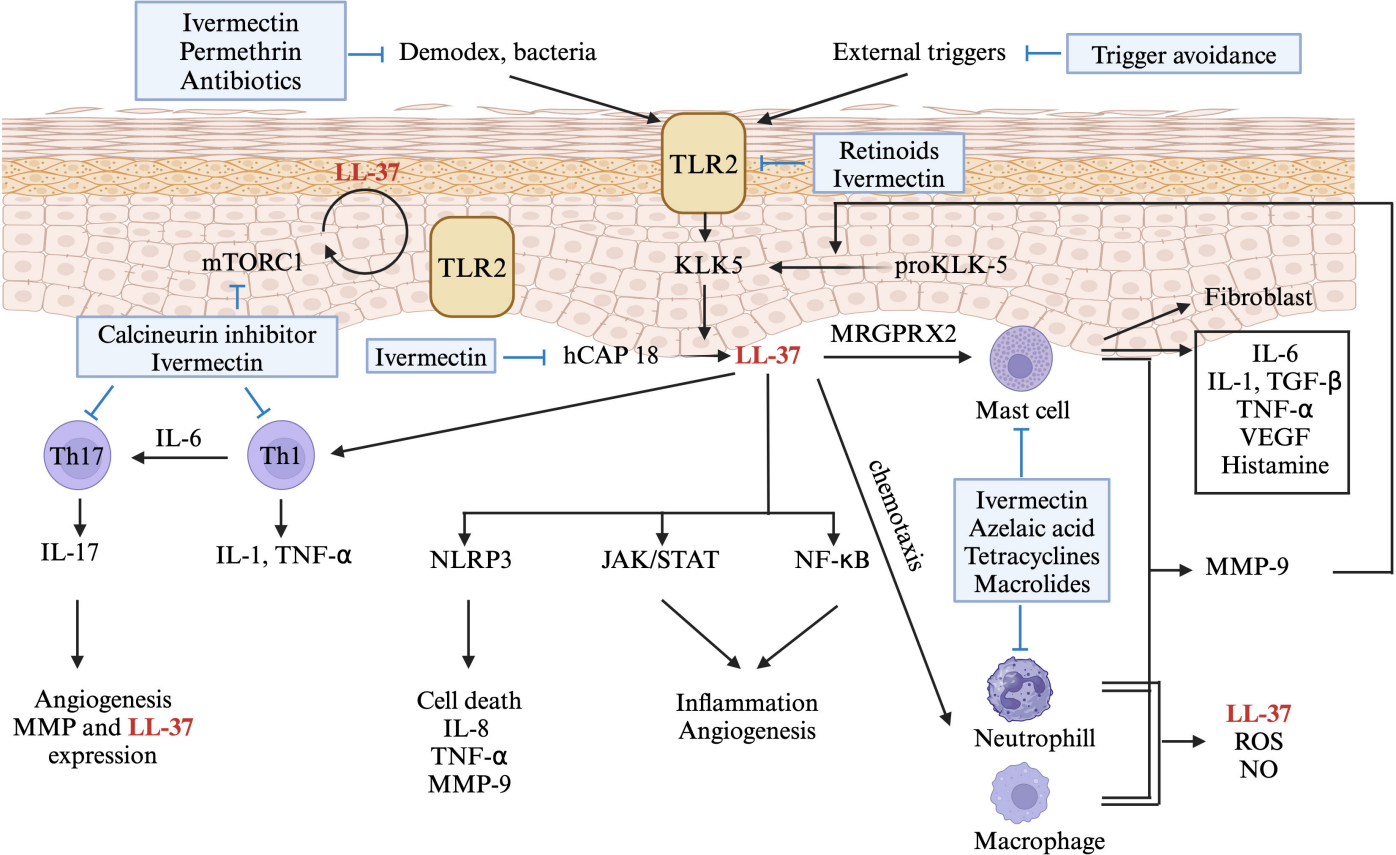
A diagram showing the role of immune dysregulation in the pathogenesis of rosacea. Environmental factors activate toll-like receptor (TLR)2 on keratinocytes, leading to kallikrein (KLK) 5 expression in rosacea. KLK5 cleaves human cathelicidin (hCAP), producing LL-37 (also known as hCAP18), triggering pathways like NLR family pyrin domain containing (NLRP)3 inflammasome, Janus protein tyrosine kinase/Signal Transducers and Activators of Transcription (JAK/STAT), and nuclear factor kappa-light-chain-enhancer of activated B cells (NF-kB). LL-37 stimulates mast cells via Mas-related G-protein coupled receptor member (MRGPR) X2, inducing the production of inflammatory cytokines and matrix metalloproteinase (MMP)9. MMP-9 further enhances KLK5 production or releases LL-37, creating a positive feedback loop. LL-37 also stimulates Th-1 and Th-17 cells, with IL-17 contributing to angiogenesis. Production of hCAP is regulated by LL-37-induced mammalian target of rapamycin (mTORC) 1 signaling, again creating a positive feedback loop. Immune-targeted therapies for rosacea are shown in blue boxes. Only drugs aligned with Swiss S1 and National Rosacea Society guidelines are indicated. This figure was created with BioRender.com and obtained authorization on February 11, 2024. TLR2, toll-like receptor 2; KLK-5, Kallikrein 5; hCAP, human cathelicidin; mTOR, mammalian target of rapamycin; IL-, interleukin; TGF-β, Transforming growth factor beta; TNF-α, tumor necrosis factor; VEGF, vascular endothelial growth factor; MRGPRX2, Mas-related G-protein coupled receptor member X2; Th-, T helper cell; NLRP3, NLR family pyrin domain containing 3; JAK/STAT, Janus protein tyrosine kinase/Signal Transducers and Activators of Transcription; NF-κB, nuclear factor kappa-light-chain-enhancer of activated B cells; MMP, matrix metalloproteinase; ROS, reactive oxygen species; NO, Nitric oxide.

Data on immune dysregulation in rosacea primarily involves disturbances in the innate immune system (**Figure 2**). Rosacea-affected skin tissues exhibit substantially higher concentrations of antimicrobial peptides, particularly cathelicidin and LL-37, than do normal skin tissues (2, 9). In addition, rosacea upregulates the expression level of TLR2, thereby increasing the secretion of KLK5 from keratinocytes. This promotes the cathelicidin activation pathway, resulting in increased LL-37 production (2, 5). These molecules play crucial roles in regulating the innate immune system in patients with rosacea. Innate immunity–related inflammatory cells such as mast cells play important roles in rosacea. While there is limited research specifically on antihistamines or mast cell stabilizers in rosacea, some studies suggest they may have a beneficial effect in reducing inflammation and symptoms (6). The proportion of mast cells is markedly increased in affected tissues. LL-37 can induce mast cells to secrete cathelicidin, which, in turn, increases the level of LL-37 (32). LL-37 can also activate neutrophils and macrophages, leading to the release of various cytokines and MMPs (33). MMPs can convert pro-KLK5 into KLK5, further increasing the level of LL-37 in affected tissues (12). These findings indicate that the innate immune system of patients with rosacea is trapped in a cycle of activation and inflammation under the effects of various triggers.

Vitamin D plays a major role in both the innate and adaptive immune systems. In particular, this vitamin regulates the expression of cathelicidin in keratinocytes (34). Increased vitamin D levels and altered *VDR* single-nucleotide polymorphisms are strongly associated with rosacea (35). It is not known whether vitamin D supplementation or toxicity leads to facial redness.

Rosacea is a chronic inflammatory skin condition. The role of the antioxidative system in the regulation of oxidative stress (e.g., during inflammation) has been implicated in the pathogenesis of rosacea (36). A study revealed marked differences between patients with rosacea and individuals without this condition in the expression levels of glutathione S-transferase polymorphisms, indicating that these polymorphisms are associated with an increased risk of rosacea (37).

In addition to the innate immune system, the adaptive immune system is affected by rosacea. Abundant Th1/Th17 cells have been observed in affected tissues (31). Th17 cells secrete interleukin (IL)-17, which induces angiogenesis through vascular endothelial growth factor and affects the expression of LL-37 in keratinocytes (38).

### 2.4 Neurovascular dysregulation and inflammation

**Figure 3:**
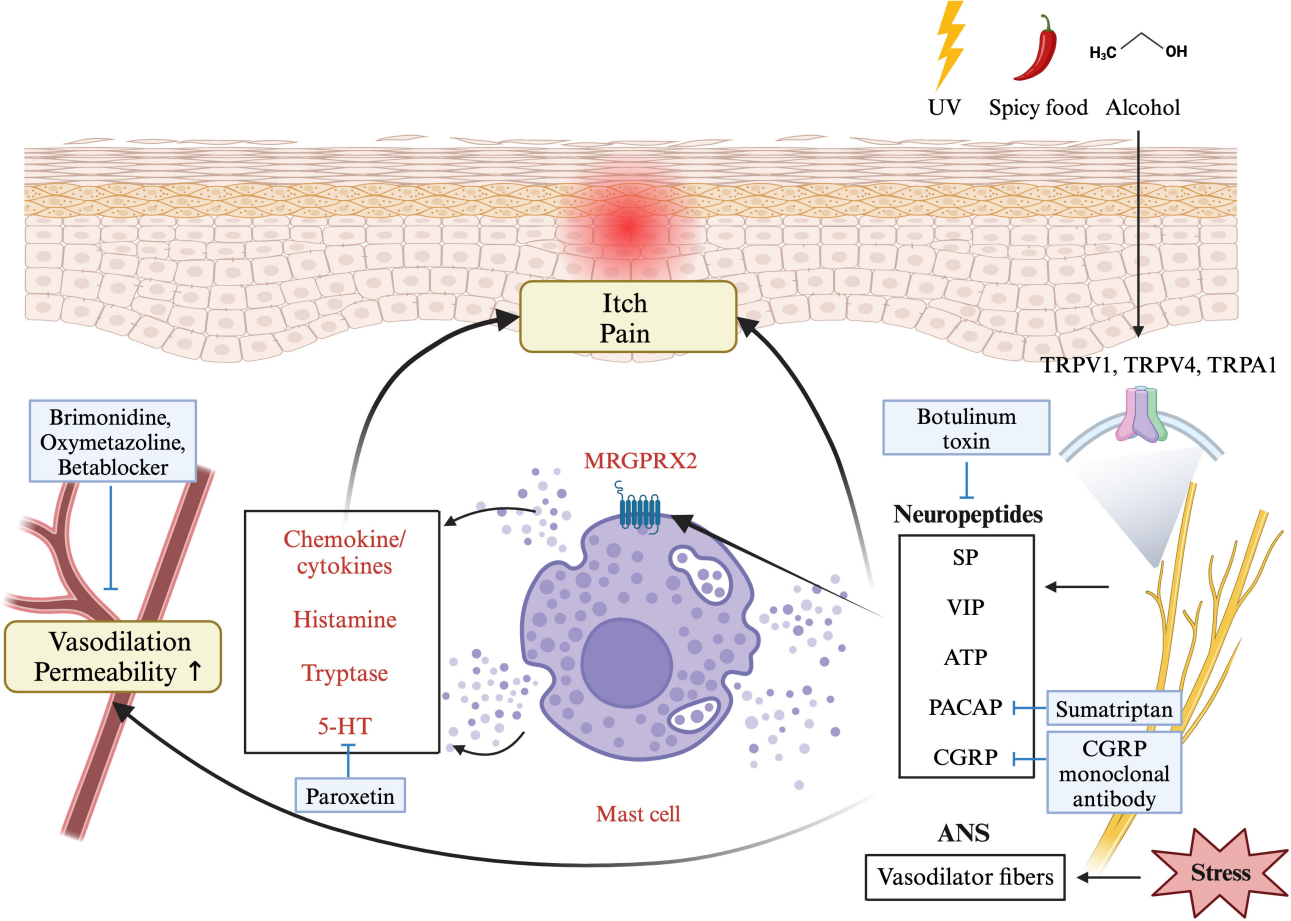
A diagram showing the pathophysiology of rosacea involving neurovascular dysregulation. External factors can activate sensory neurons via transient receptor potential cation channel (TRPV) receptors, leading to neuropeptide release. This activates mast cells through Mas-related G-protein coupled receptor member (MRGPR) X2, causing degranulation of histamine, serotonin, and chemokines, resulting in vasodilation, pain, and itch. Stress causes the autonomic nervous system to overstimulate vasodilator fibers, affecting the layer of smooth muscle enveloping the blood vessels, ultimately resulting in vasodilation. The figure includes medications recommended by Swiss S1 and National Rosacea Society guidelines (blue boxes) associated with this mechanism. This figure was created with BioRender.com and obtained authorization on July 03, 2024. UV, ultraviolet; TRP-, transient receptor potential channel; SP, substance P; VIP, vasoactive intestinal polypeptide; ATP, adenosine triphosphate; PACAP, pituitary adenylate cyclase-activating peptide; CGRP, calcitonin gene related peptide; ANS, autonomic nervous system.

Transient receptor potential (TRP) channels, including TRPV1, TRPV4, and TRPA1, play key roles in neurovascular dysregulation (**Figure 3**). These TRPs are expressed not only in nerve endings but also in the endothelium and keratinocytes (27). External factors such as cold, heat, alcohol, spicy foods, and chemicals can theoretically activate these TRPs, leading to the release of vasoactive neurotransmitters, such as PACAP, CGRP, vasoactive intestinal peptide, and substance P (39). These neurotransmitters result in vasodilation and stimulate downstream inflammatory cells such as mast cells, macrophages, and neutrophils, thereby inducing inflammatory response (3, 27). Neuropeptides released from sensory neurons affect vascular endothelial cells, causing vasodilation and increasing capillary permeability (27, 40). In addition, these neuropeptides activate cutaneous mast cells through MRGPRX2, leading to the degranulation of these cells and the production of chemokines and cytokines (27, 40), thereby exacerbating rosacea.

Several studies suggest that adenosine 5’-triphosphate (ATP), a purinergic nucleotide and neurotransmitter, also participates in rosacea pathogenesis by regulating vascular tone and immune responses (41). In human microvascular endothelial cells (HMEC-1), an ATP analogue enhances the secretion of pro-inflammatory cytokines such as IL-6 and IL-8, promotes the release of chemokines like CCL-2, CCL-5, CCL-21, and CXCL1, and increases the expression of adhesion molecules (41). These actions indicate that ATP may exacerbate inflammation and vasodilation, which are key components in rosacea.

Rosacea patients exhibit heightened responses to heat or stress, leading to increased sweating and cutaneous vasodilation (42). This involves sympathetic nerve excitation, affecting vascular smooth muscle contraction in superficial peripheral vessels through multiple alpha-receptors (43). Additionally, rosacea may feature dilated blood and lymphatic vessels, contributing to erythema and edema (2). Patients with neurogenic rosacea, which resembles small fiber neuropathy, often present with gastrointestinal symptoms and dysautonomia (44). Both conditions share similar manifestations, complications, and treatments, indicating common autonomic nervous system dysregulation (44).

The aforementioned hypothesis remains to be confirmed through studies on the associations of these vasoactive neurotransmitters with rosacea. An *in vitro* study revealed that mast cells require prior upregulation of TRPV4 for effective LL-37-mediated stimulation and subsequent complete degranulation (45). This indicates a possible crosstalk between neurovascular dysregulation and immune dysregulation—two major pathogenic mechanisms underlying rosacea (32).

## 3 Current therapeutic strategies and clinical trials

Currently, treatment options for rosacea include topical and oral drugs, light devices, and appropriate skincare and lifestyle management (46).

This section provides an overview of various treatment strategies for rosacea particularly focused on modulating the immune system either directly or indirectly. To highlight key therapeutic targets, the strategies were categorized on the basis of their mechanisms of action. Although a single medication may affect multiple mechanisms, we focused on the primary target of each strategy. The following sections present commonly recommended treatment options with an overview of emerging treatment approaches based on The Oxford 2011 Levels of Evidence (47) (**Supplementary Tables 1-4**). Medications that have been approved by the US Food and Drug Administration (FDA) are marked with an asterisk (*).

Laser, light therapies, and surgery are essential for treating rosacea-associated telangiectasia resistant to standard therapies. Treatments such as potassium titanyl phosphate (KTP) laser, pulsed dye laser (PDL), neodymium-doped yttrium aluminum garnet (Nd : YAG) laser, and intense pulsed light (IPL) therapies address telangiectasis, erythema, and flushing (48). However, their impact on immune dysregulation and neuroinflammation lacks evidence and is beyond this article’s scope.

### 3.1 Immune-targeted therapy

Immune dysregulation in rosacea involves innate immune disturbances, including TLR2 activation, KLK5 release, and cathelicidin cleavage into LL-37 (9). These processes, triggered by UV radiation and pathogens, are crucial in rosacea inflammation (5, 6). Adaptive immunity, especially Th1/Th17 cells, is also affected in rosacea (31), indicating a complex immune response. **Supplementary Table 2** lists selected therapeutics targeting immune dysregulation for rosacea. FDA-approved anti-inflammatory agents for rosacea include oral tetracyclines (49), topical minocycline (50), and azelaic acid (51, 52). Off-label, oral isotretinoin (53, 54) and topical calcineurin inhibitors (55, 56) are widely used. Studies have explored other potential anti-inflammatory agents, such as secukinumab, targeting IL-17, due to data indicating Th17 cells’ role in rosacea.

#### 3.1.1 Oral tetracyclines as anti-inflammatory agents

Oral doxycycline (40 mg/day; 30 mg immediate release and 10 mg delayed release) is a well-established, FDA-approved treatment option for rosacea (57). Its benefits have been validated by a phase III randomized controlled trial (49). Submicrobial doses of doxycycline can effectively alleviate inflammation by suppressing the production of proinflammatory cytokines, reactive oxygen species, nitric oxide synthetase, and MMPs, while also avoiding antibiotic resistance or dysbiosis (58, 59). Furthermore, doxycycline can inhibit the cathelicidin activation pathway and immune cell recruitment (59). These anti-inflammatory effects of doxycycline have led to its recommendation by treatment guidelines (46, 60). While tetracyclines are commonly used in rosacea and acne for their anti-inflammatory effects, their antimicrobial properties likely alter the composition of the skin microbiome. A systematic review found that these treatments mostly decrease *Cutibacterium acnes* and increase the alpha diversity of the cutaneous microbiota (61).

Other tetracyclines also demonstrate significant anti-inflammatory effects, including the inhibition of chemotaxis, granuloma formation, and proteases (62). Therefore, various tetracycline drugs, formulations, and dosages are commonly used off-label in rosacea treatment (62). These alternatives include tetracycline (250–1000 mg/day), doxycycline (100–200 mg/day), and minocycline (100–200 mg/day). These agents are effective against PPR (59, 63, 64).

Other forms of oral tetracyclines have recently shown efficacy in treating rosacea. DFD-29, a minocycline extended-release oral capsule (40 mg), exhibited a significantly higher level of efficacy than did placebo and doxycycline (40 mg) in the treatment of PPR with the plasma level of drug was maintained below its antimicrobial threshold (65).

A new generation tetracycline, sarecycline, has proven effective and safe in the treatment of PPR. Compared with control, sarecycline significantly improved patients’ IGA scores, reduced inflammatory lesion counts, and alleviated rosacea’s secondary symptoms such as burning sensation, without causing any adverse effects (66). While not extensively examined in humans, sarecycline has demonstrated anti-inflammatory activity comparable to doxycycline and minocycline in a rat paw edema model (67).

Notably, the mechanism of action of tetracyclines in rosacea primarily involves modulating immune dysregulation, not neurovascular dysregulation. Therefore, when using tetracyclines in patients with ETR, significant improvement is generally not expected (46). Tetracycline may inhibit the proinflammatory process induced by the ATP analogue in vascular endothelial cells *in vitro* (41, 68). While there may be a slight effect on persistent erythema, the level of evidence supporting this is not strong (46).

#### 3.1.2 Topical azelaic acid

Azelaic acid gel (15%) is a well-established, FDA-approved topical agent. According to standard guidelines and multiple randomized controlled, double-blinded multicenter trials, azelaic acid is beneficial against mild to moderate inflammatory papules or pustules (46, 57, 60, 69, 70). However, it exhibits a low level of efficacy against erythema, primarily in patients with the papulopustular phenotype (51, 52). Azelaic acid works by suppressing KLK5 and cathelicidin expression and activating peroxisome proliferator-activated receptor γ, thus reducing the degrees of inflammatory responses and the levels of proinflammatory factors such as IL-1, IL-6, and TNF-α (71, 72). Additionally, this medication can suppress the UV-induced activation of nuclear factor (NF)-κB p65 subunit (73).

#### 3.1.3 Topical tetracyclines

Minocycline foam 1.5% (FMX103), which was approved by the US FDA in 2020, was found to be effective and safe against moderate to severe papulopustular rosacea (PPR) in two 12-week, randomized, double-blinded, vehicle-control phase III trials (50). Another topical formulation is minocycline gel, whose 1% and 3% concentrations were tested in a 12-week, randomized, double‐blinded, vehicle‐controlled phase IIb trial; the results revealed that both concentrations, particularly the 3% concentration, significantly reduced inflammatory lesion counts and improved the Investigator’s Global Assessment (IGA) scores (74). As mentioned above, oral minocycline act as anti-inflammatory agents. While topical minocycline shows promise in delivering anti-inflammatory effects with reduced risk of systemic adverse effects, further evaluation is needed.

#### 3.1.4 Oral isotretinoin

Isotretinoin is an off-label treatment option for rosacea. It is indicated for moderate to severe PPR (46, 57, 60). Isotretinoin is typically administered at a low daily dose (0.25–0.3 mg/kg) over a 4-month period; this is followed by a gradual dose reduction (54). Mechanistically, isotretinoin works by downregulating TLR2 expression in keratinocytes (53, 54). By inhibiting the activity of the sebaceous gland and reducing the production of sebum, isotretinoin can delay the progression of inflamed phyma when used during the prefibrotic phase of PPR (54, 75). Although the anti-inflammatory effect of isotretinoin might theoretically benefit erythema by inhibiting neuro- inflammation, its efficacy in treating erythema in rosacea patients shows inconsistent results (76). Some studies indicate that low-dose (0.25 mg/kg/day) isotretinoin is not effective in treating erythema and telangiectasia (77), while others suggest that an intermediate dose (20 mg/day) effectively improves erythema within four weeks (78).

#### 3.1.5 Oral macrolides

Macrolide drugs, such as erythromycin, clarithromycin, and azithromycin, are secondary agents used in the systemic treatment of rosacea. For azithromycin, the dosage is 500 mg three times weekly for 4 weeks, followed by 250 mg three times weekly for additional weeks. For clarithromycin, the dosage is 250 mg twice daily for 4 weeks, followed by 250 mg once daily for the next 4 weeks. They are particularly useful for patients with contraindications to tetracycline, such as those who are refractory or pregnant (57, 79). The therapeutic effects of macrolides extend beyond their antimicrobial properties to include immunomodulatory effects. For example, azithromycin can modulate transcription factors such as NF-κB, reduce the release of inflammatory cytokines, impede the migration of neutrophils, inhibit the activation of neutrophils and eosinophils, and suppress the release of reactive oxygen species (57, 80). However, despite the low level of evidence for the efficacy of azithromycin in treating rosacea, its gastrointestinal adverse effects are increasingly becoming a concern (81, 82).

#### 3.1.6 Hydroxychloroquine

Hydroxychloroquine effectively suppresses the activation of mast cells by inhibiting Ca^2+^-activated K+ channels, leading to a reduction in local Ca^2+^ influx (83). This suppression results in the inhibition of inflammatory factor release, chemotaxis, and degranulation, ultimately reducing chemokine production and neutrophil and monocyte recruitment (83).

Patients who received hydroxychloroquine for 8 weeks exhibited improvements in their rosacea phenotypes along with improved IGA and Clinician Erythema Assessment (CEA) scores, indicating a tendency toward symptomatic relief (83). In a separate multicenter, randomized, double-blind, double-placebo pilot study, participants received either oral hydroxychloroquine (200 mg twice daily) or doxycycline (100 mg once daily) along with their respective placebos for 8 weeks; in this study, hydroxychloroquine was found to be noninferior to doxycycline in terms of rosacea-specific quality-of-life scores (84).

#### 3.1.7 Secukinumab

IL-17, a proinflammatory cytokine produced by Th17 cells, has been implicated in rosacea-related inflammation (31, 85). Th17 activation contributes to the upregulation of LL-37 expression, which, in turn, promotes the production of cathelicidin (85). The synergistic actions of LL-37 and IL-17 result in the release of C-X-C motif chemokine ligand 8 and IL-6, facilitating neutrophil chemotaxis and Th17 differentiation, respectively (85). This Th1/Th17 polarized inflammation, often underrecognized, is common across all subtypes of rosacea (31). In an exploratory study having an open-label, rater-blinded design, secukinumab, an IL-17A inhibitor, significantly reduced papule counts and improved global severity and median RosaQOL scores after 16 weeks of systemic therapy (86). However, more comprehensive and definitive evidence from high-quality randomized controlled trials is needed to fully understand the efficacy of secukinumab in the management of rosacea.

#### 3.1.8 Janus kinase inhibitors

The JAK-STAT signaling pathway plays a pivotal role in the proinflammatory processes within immune cells. In the pursuit of effective rosacea treatments, oral JAK inhibitors like tofacitinib and abrocitinib have been explored (87, 88). A compelling case series involving 21 patients with erythematotelangiectatic and papulopustular rosacea demonstrated promising results, with 71.4% experiencing significant regression of facial erythema (IGA ≤ 1) and a mean change of -2.24 in the IGA score (87). Additionally, a separate case series highlighted the efficacy of abrocitinib in improving steroid-induced rosacea (88). These findings underscore the substantial potential of JAK inhibitors for broader utilization in the management of rosacea.

#### 3.1.9 Topical calcineurin inhibitors

Pimecrolimus cream proves to be an effective treatment option for those with mild to moderate inflammatory rosacea (55). Pimecrolimus exhibits anti-inflammatory activity against PPR by inhibiting the activation of T cells, suppressing the production of inflammatory cytokines, and preventing the release of cytokines from T and mast cells through the inhibition of calcineurin phosphatase (89). The calcineurin inhibitor tacrolimus can treat erythema on rosacea patients (90). However, a higher level of evidence is available for 1% pimecrolimus than for tacrolimus (55, 56, 60). Prescribing these drugs should be done cautiously due to the potential risk of causing rosacea-like eruptions (91). The potential adverse events may be attributable to their immunosuppressive effects, which results in the overgrowth of microorganisms such as *D. folliculorum* (91). Therefore, pimecrolimus and tacrolimus may be regarded as dual-edged swords in the treatment of rosacea (91).

#### 3.1.10 Mammalian target of rapamycin inhibitor

Mammalian target of rapamycin complex 1 (mTORC1) can regulate cathelicidin through a positive feedback loop (92). In this loop, LL-37 activates mTORC1 signaling by binding to TLR2, thereby upregulating the expression of cathelicidin in keratinocytes (92). Excess LL-37 may induce NF-κB activation through mTORC1 signaling, leading to the production of disease-specific cytokines and chemokines (92). Overexpression of TLR7 in keratinocytes stimulates the mTORC1 pathway through NF-κB signaling (93). In a pilot study involving 18 women with rosacea, the patients were randomized to receive either a placebo (n = 8) or 0.4% topical rapamycin (sirolimus) ointment (n = 10) for 4 weeks (92). After the intervention, the level of clinical improvement was significantly higher in the rapamycin group than in the placebo group. Topical treatment with rapamycin significantly reduced the patients’ CEA and IGA scores (92).

#### 3.1.11 Artemisinin

Artemisinin, a widely recognized antimalarial drug, effectively suppressed the infiltration of CD4+ T cells in a mouse model of LL-37-induced rosacea (94, 95). This drug also suppressed the LL-37-induced invasion of neutrophils and macrophages, thereby mitigating innate immune responses against rosacea (95). In HaCaT cells, artemisinin suppressed the release of proinflammatory factors, potentially through the NF-κB pathway (95). An open-label trial involving 130 patients with rosacea who received artemether emulsion, one of the most extensively studied lipid-based derivatives of artemisinin, revealed markedly reduced papule and pustule scores as early as 4 weeks into treatment; notably, the erythema scores of patients treated with artemether emulsion were similar to those of patients treated with standard metronidazole for 4 weeks (96).

#### 3.1.12 ACU-D1

The 26S proteasome, a protein complex responsible for NF-κB degradation, regulates the activation of NF-κB. Therefore, inhibiting the 26S proteasome can result in the inhibition of NF-κB; this offers a novel target for rosacea treatment. A two-arm, vehicle-controlled trial evaluated the efficacy of a novel proteasome inhibitor, topical ACU-D1—pentaerythritol tetrakis (3-(3, 5-di-tert-butyl-4-hydroxyphenyl) propionate). The trial included 27 patients in the active arm and 12 patients in the vehicle arm, all presenting with moderate to severe PPR. ACU-D1 was found to be safe and well-tolerated by the patients; it effectively reduced inflammatory lesions and erythema in the patients. Although this trial lacked the statistical power to determine between-arm significance, the evaluation of both efficacy and safety yielded favorable results (97).

#### 3.1.13 Dapsone

Dapsone exerts its anti-inflammatory effects by inhibiting the production of ROS, mitigating the effects of eosinophil peroxidase on mast cells, and suppressing inflammatory responses mediated by neutrophils (98). A case report indicated sustained remission of granulomatous rosacea after the systemic administration of dapsone (dose: 100 mg/day) (99). A double-blind, randomized clinical trial involving 56 adults with PPR demonstrated that 5% dapsone gel exhibited efficacy similar to that of 0.75% metronidazole gel in improving IGA scores and reducing the lesion counts (100). The first to study dapsone use on ETR, reported that 5% dapsone gel used alone for 12 weeks showed significant improvement in the Investigators Global Assessment score, burning sensation, pruritus, edema, and erythema (101).

#### 3.1.14 ϵ-Aminocaproic acid

ϵ-Aminocaproic acid can effectively inhibit trypsin-like proteases, including KLK5, which regulates the cleavage of the cathelicidin precursor protein into LL-37 (102). In a randomized, double-blind, placebo-controlled study involving 11 patients with PPR, no significant differences in the IGA or CEA scores were noted between the treatment and placebo groups at any time point. Notably, in the treatment group, these scores were significantly improved in week 12 compared with baseline (102).

#### 3.1.15 Tranexamic acid

Tranexamic acid (TXA), an antifibrinolytic drug used to treat bleeding conditions, functions by inhibiting the conversion of plasminogen to plasmin, thus suppressing plasmin-induced angiogenesis (103). This suppression is achieved through the reduction of CD31^+^ microvessel counts and the downregulation of vascular endothelial growth factor expression (104). In the context of skin inflammation, TXA inhibits the production of TLR2, proinflammatory cytokines (IL-6 and TNF-α), and chemokines in keratinocytes primed by LL-37 (104). In an unblinded study, TXA solution–infused wet dressing and microneedling with TXA solution ameliorated the symptoms of erythematotelangiectatic rosacea (103). In a retrospective study investigating the effects of intradermal microinjections of TXA on erythematotelangiectatic rosacea, the observed improvements in symptoms persisted for 3 months (105). In a randomized, vehicle-controlled, split-face study, topical TXA enhanced the barrier function of the epidermal layer and alleviated the clinical signs of rosacea (106). These effects are attributed to the inhibition of protease activated receptor 2 activation, which reduces the calcium influx (106). For oral TXA, one study divided patients into two groups. Seventy patients were treated with doxycycline therapy plus oral TXA or doxycycline therapy alone for 8 weeks. The results showed that the TXA group had better outcomes compared to doxycycline (107).

### 3.2 Targeted neurovascular therapy for inflammation

Neurovascular dysregulation in rosacea may be linked to immune dysregulation (3, 27). Activation of TRP channels and release of vasoactive neurotransmitters trigger vasodilation, inflammation, and mast cell degranulation, contributing to the symptoms (40). **Supplementary Table 3** lists selected therapeutics targeting neurovascular dysregulation for rosacea. FDA-approved treatments for inflammation include topical brimonidine and oxymetazoline (46), acting as vasoconstrictors. Off-label, oral beta blockers are widely used (108). Pilot studies on agents like paroxetine (109), sumatriptan (110), and monoclonal antibodies against CGRP (28) show promise, suggesting a novel treatment approach targeting vasoactive neurotransmitters in rosacea.

#### 3.2.1 Brimonidine

Among drugs targeting neurovascular dysfunction in rosacea, two have received FDA approval (46). The first one is brimonidine topical gel (0.33%), which is a vasoconstrictive alpha-2 adrenergic receptor agonist acting on the microvascular smooth muscles of the facial skin (46). Brimonidine specifically addresses facial erythema instead of papules and pustules (60). Clinical trials ranging in duration from 1 month to 12 months have consistently demonstrated that topical brimonidine is both safe and effective as a maintenance therapy for rosacea (111, 112). A short-term multicenter, randomized study demonstrated its efficacy in improving moderate rosacea (at least a 1-grade improvement) (113). However, brimonidine is not effective in managing telangiectasia (57, 60). Brimonidine had significant risk of developing adverse effects include local erythema, pruritus, burning sensation, and exacerbated flushing symptoms (57, 60, 114).

#### 3.2.2 Oxymetazoline

Oxymetazoline is the second FDA-approved vasoconstrictor for the topical management of persistent erythema in patients with rosacea (46). As a sympathomimetic agent, oxymetazoline acts as a selective alpha1A adrenoreceptor agonist, exhibiting vasoconstrictive and anti-inflammatory properties (57). Multiple phase III clinical trials have reported that 1% oxymetazoline hydrochloride cream, administered for either 29 days or 52 weeks, was well-tolerated, safe, and effective in treating persistent facial erythema in patients with rosacea (115–117). Only a small proportion of patients experienced adverse effects such as apparent worsening of facial erythema, rebound effects, and occasional paradoxical erythema (115–117). A meta-analysis of adverse effects found that using oxymetazoline, compared to the vehicle, had a significantly higher risk of application-site dermatitis (RR = 8.91, 95% CI: 1.76–45.23), while other adverse effects did not show significant statistical differences (118). These findings highlight the differences between oxymetazoline and topical brimonidine in terms of safety and efficacy (114).

#### 3.2.3 β-blocker

The potential mechanism of action of non-selective beta-blockers lies in the inhibition of beta-2 receptors on cutaneous blood vessels, reducing vasodilation (108). Additionally, systemic administration exhibits a reduction in sympathetic tone, thereby alleviating patient anxiety (119). A systematic review indicates that carvedilol and propranolol are effective for rosacea patients with persistent facial erythema and flushing (108). For carvedilol, the dosage ranges from 6.25 to 37.5 mg daily, which can be divided into doses such as 6.25 mg twice daily. For propranolol, the dosage is typically 30-40 mg daily. In a randomized controlled trial, carvedilol demonstrated superior efficacy compared to brimonidine in addressing telangiectasia and facial erythema (120). Moreover, carvedilol significantly improved patients’ depression or anxiety status (120). While beta-blockers offer alternative therapeutic options for rosacea, it is essential to be cautious about common side effects such as hypotension, bradycardia, bronchospasm, and dizziness (121).

#### 3.2.4 Paroxetine

Treatment with paroxetine, a potent selective serotonin (5-HT) reuptake inhibitor, led to improvements in CEA scores, flushing, and burning sensation in a multicentered, randomized, double-blinded, and placebo-controlled trial involving patients with rosacea (109). Paroxetine modulates 5-HT uptake, and dysfunctional 5-HT regulation may result in abnormal blood vessel dilation and constriction (2, 122, 123). Mast cells and platelets were also proved to release 5-HT in several inflammatory skin diseases (124, 125). An association has been reported between rosacea and a single-nucleotide polymorphism of the 5-HT2A receptor (126).

#### 3.2.5 Sumatriptan

PACAP is a potent vasodilator that directly affects smooth muscle cells in arterioles (127). A study reported elevated levels of PACAP expression in rosacea-affected tissues, suggesting its potential relevance in TRPV-mediated edema (29). Another study utilizing both clinical and experimental models confirmed that PACAP38 induces sustained flushing and facial edema (110). In a double-blind, randomized, placebo-controlled, crossover trial, sumatriptan alleviated these symptoms (110). Sumatriptan acts as a 5-HT1B/1D receptor agonist, inhibiting mast cell degranulation and reducing PACAP levels (128, 129). A case report suggested that oral sumatriptan (50 mg) successfully treated a patient with severe and painful rosacea, markedly reducing burning sensation, swelling, redness, and pain (130).

#### 3.2.6 Monoclonal antibodies against CGRP

Erenumab, galcanezumab, fremanezumab, and eptinezumab are four FDA-approved monoclonal antibodies that target CGRP. These antibodies are primarily used for preventing migraine (131, 132). Another novel oral CGRP inhibitor in the pipeline for chronic migraine is atogepant (133). CGRP is involved in the pathophysiology of migraine through nociceptive processes in the trigeminovascular system (134). Notably, CGRP serves as a vasoactive neurotransmitter in rosacea, causing vasodilation and inducing inflammatory response (3, 135). In 2020, an exploratory trial was initiated to investigate the efficacy and tolerability of erenumab in the preventive treatment of persistent redness and flushing associated with rosacea; this trial was anticipated to conclude in 2021 (136). Furthermore, an exploratory comparative case series involving 13 patients explored the benefits of CGRP monoclonal antibodies for patients with both rosacea and migraine (28). The results revealed marked posttreatment improvements in the patients’ severity scores (28). However, two patients experienced dermatitis at the injection site (28). Although most patients in this study experienced improvements in rosacea, large-scale randomized clinical trials are still needed to confirm the efficacy and safety of CGRP monoclonal antibodies.

#### 3.2.7 Botulinum toxin

Botulinum toxin (BTX) improves neurovascular dysfunction in rosacea by blocking the release of neurotransmitters, such as acetylcholine, calcitonin gene-related peptide, vasoactive intestinal peptide, substance P, and glutamate, or by reducing non-noxious stimulation (135, 137, 138). This action inhibits the skin’s vasodilator system (stimulated by peripheral autonomic nerves), thereby suppressing rosacea-associated neuroinflammation and vasodilation (4, 139). BTX inhibits the activation of TRPV1 by disrupting the lipid raft activity associated with TRPV1 through structural and functional interactions with it (140). Moreover, BTX directly blocks the degranulation of mast cells by cleaving SNARE protein in these cells (141). In a randomized, controlled, split-face study involving 22 patients, one side of the cheek was treated with a combination of BTX and broadband light. Compared with the pretreatment condition and control groups, the experimental group exhibited reduced global flushing symptom scores, erythema index scores, transepidermal water loss, and sebum secretion (142). The effects of using BTX typically appear within 1-2 weeks and can last for 3-6 months. The most common side effect was localized pain, while the rarer and more concerning side effect was paralysis of motor muscles, with a pooled incidence of 4.3% (95% CI: 1.8 - 7.8%). Most cases of paralysis resolved on their own (143).

### 3.3 Targeted antimicrobial therapy

Research on the cutaneous microbiome in rosacea focuses on *Demodex folliculorum*, *Staphylococcus epidermidis*, *Bacillus oleronius*, *Bartonella quintana*, and *Chlamydia pneumoniae* (5, 8, 13, 14). Demodex, with its chitin exoskeleton, triggers inflammation by increasing TLR2 levels (5, 8), and therefore becomes the major target of antimicrobial therapy for rosacea. **Supplementary Table 4** lists therapeutics targeting microbiota for rosacea. FDA-approved antimicrobials include topical metronidazole (82, 144), ivermectin (145), and microencapsulated benzoyl peroxide (146). Off-label, oral metronidazole (147), oral ivermectin (148), topical sulfur preparation and topical permethrin (148) target Demodex as well. Ongoing studies investigate topical omiganan (149) and oral rifaximin (150). Despite optimism for treatments like bacterial transfer, probiotics, or prebiotics to balance the microbiome, the lack of large-scale trials hampers confirming their efficacy (151).

#### 3.3.1 Metronidazole: oral and topical uses

Because of its dual efficacy as an antibiotic and antiparasitic agent, FDA-approved topical metronidazole is widely used for the treatment of rosacea across the globe and indirectly reduces inflammation by putative reduction of *Demodex* colonization. *Demodex* mites are implicated in the pathogenesis of rosacea, and the antiparasitic effect of metronidazole plays a key role in this context (57). Metronidazole’s efficacy in rosacea treatment is also attributed to its anti-inflammatory, immunosuppressive, and antioxidative characteristics, involving the reduction of reactive oxygen species production from neutrophils and inhibition of IL-17 (152–154). The efficacy of topical metronidazole is supported by clinical trials, including randomized, placebo-controlled double-blind studies, as highlighted in a Cochrane review (82, 144). Clinical guidelines recommend topical metronidazole for the treatment of PPR (46, 57, 60). The efficacy of oral metronidazole was evaluated in a double-blind trial, where a combination therapy comprising metronidazole (200 mg twice a day) and 1% hydrocortisone cream was administered for 6 weeks; this therapy reduced the severity of rosacea in 10 out of 14 patients (147).

#### 3.3.2 Ivermectin: oral and topical uses

The US FDA have approved 1% ivermectin cream for the treatment of PPR (155). Clinical guidelines recommend topical ivermectin as a first-line treatment for rosacea, citing high-level evidence for its efficacy in addressing papules and pustules—the key features of rosacea (46, 60). A 2016 network meta-analysis identified ivermectin 1% cream to be the most effective treatment option for inflammatory papules and pustules (145). Regarding the Demodex decrease rate, both topical and systemic ivermectin are superior to other anti-Demodex therapies, achieving nearly a 100% decrease rate (156). Its therapeutic mechanism involves effectively reducing *Demodex* infestation through neurotoxic activity (157). Owing to its anti-inflammatory properties, ivermectin reduces the levels of IL-1b and TNF-α while increasing the level of IL-10. Additionally, ivermectin reduces the expression of proinflammatory genes such as IL-8, LL-37, human β-defensin 3, and TNF-α (158). At the protein level, significant reductions were observed in the levels of LL-37 and IL-8 (158).

#### 3.3.3 Sulfur preparation

The US FDA-approved topical treatment also included sulfur preparations in various formulation such as creams, gels and cleansers. The most common formulation is sodium sulfacetamide, 10% (46). Sulfur preparations have shown positive effects on both erythematotelangiectatic rosacea (ETR) and papulopustular rosacea (PPR), similar to metronidazole (159). This effect may be due to the significant reduction in Demodex count with the sulfur-sodium combination (160). However, sulfur has a more noticeable irritating effect compared to metronidazole, permethrin, lindane, and crotamiton (161).

#### 3.3.4 Microencapsulated benzoyl peroxide cream

Benzoyl peroxide, a potent oxidizing agent, is commonly used in acne treatment (155) and indirectly reduces inflammation through putative antibacterial effects. In 2022, the US FDA approved encapsulated cream benzoyl peroxide (E-BPO) 5% for patients with rosacea (155). The advantage of this silica-based encapsulated formulation is that it facilitates a gradual release of benzoyl peroxide into the skin. This approach enhances efficacy and prevents skin irritation associated with traditional bolus formulations of benzoyl peroxide, thereby improving tolerability (155, 162). Although E-BPO’s mechanism of action in the treatment of rosacea remains hypothetical, it primarily centers around the antibacterial effect of this drug (136, 163). In two 12-week, identical, parallel, randomized, double-blind phase III trials, a high proportion of E-BPO-treated patients achieved a clear/almost clear status in the IGA and a reduced lesion count (146). However, a few adverse events were noted; these included pain, erythema, pruritus, and edema (146). Furthermore, a 52-week, single-arm phase III trial investigating the long-term use of E-BPO indicated that prolonged E-BPO use is a safe and well-tolerated therapeutic approach (164).

#### 3.3.5 Topical permethrin

The role of *Demodex* mites in the development of rosacea is well-established and its anti-inflammatory effect is indirect though putative reduction of mites that may stimulate the immune system. Researchers have explored permethrin, an antiparasitic belonging to the pyrethrin group, as a treatment choice for rosacea (154). The results indicated a notable decrease in the density of *Demodex* mites, indicating permethrin as a primary treatment choice for *Demodex* infestation (165). In a 12-week, double-blind study involving 20 patients with PPR, the application of 5% permethrin cream resulted in a marked reduction in the density of *Demodex* mites and an improvement in the symptoms of rosacea compared with the effects of a cream base (140). However, the use of permethrin for the treatment of rosacea is currently considered off-label.

#### 3.3.6 Omiganan topical gel

Omiganan is an anti-microbial peptide tested against rosacea. In randomized, double-blind, vehicle-controlled, parallel-group, multicenter phase III trial involving patients with severe PPR, topical application of 1.6% omiganan gel significantly outperformed the vehicle in reducing mean inflammatory lesion counts and improving IGA scores in week 12 compared with baseline (149). The underlying mechanism of action involves the rapid bactericidal and fungicidal effects of omiganan against a wide range of pathogens (166).

#### 3.3.7 Rifaximin

Rifaximin, a non-absorbed, gut-active antibiotic with broad-spectrum efficacy, is used to treat small intestinal bacterial overgrowth (SIBO) (155). Studies have indicated a higher prevalence of SIBO among patients with rosacea than in the normal population (167, 168). Administering rifaximin to these patients not only mitigate SIBO but also ameliorate rosacea symptoms. The underlying mechanism suggests that SIBO disrupts immunity, triggering rosacea by increasing the levels of TNF-α or other cytokines, reducing the level of IL-17, and stimulating Th1-mediated immune response (150). In a prospective trial involving 113 patients, a 10-day treatment rifaximin (1200 mg/day) significantly reduced the number of cutaneous lesions compared with the findings in the control group (150). Nonetheless, further research is needed to confirm the efficacy of rifaximin. In 2022, a phase IIa, multicenter, double-blind, placebo-controlled, randomized clinical trial (NCT 05150587) investigated the efficacy and safety of extended-release rifaximin against moderate to severe rosacea (169). The research results are yet to be published.

## 4 Promising therapeutic strategies in preclinical study with anti-inflammatory activity

Several therapeutic strategies for rosacea show promise in pre-clinical studies (**Supplementary Table 5**). Recent years have seen a growing interest in the potential of herbal medicines against rosacea. Compounds such as osthole, celastrol, *Coptis chinensis* Franch, and paeoniflorin have demonstrated efficacy in animal or cellular models by inhibiting inflammatory pathways, thereby alleviating rosacea symptoms (170–172). In an animal model of LL-37-induced rosacea-like lesions, the use of erythroid differentiation regulator 1 mitigated erythema, inflammatory cell infiltration, and microvessel density (173). The same model was used for benvitimod—an aryl hydrocarbon receptor agonist; upon stimulation, benvitimod inhibited TLR2-induced inflammatory responses (174). Furthermore, this agonist significantly ameliorated the rosacea-like symptoms (174).

Among antidiabetic medications, both metformin and pioglitazone have gained prominence. Pioglitazone, which is delivered using a new nan-oemulsion formulation, enhances the drug–skin contact and dermal retention while exhibiting anti-inflammatory activity (175). Metformin is effective in improving rosacea-like lesions, reducing LL-37- and TNF-α-induced reactive oxygen production, and mitigating mitogen-activated protein kinase/NF-κB signal activation in keratinocytes (176).

Melatonin, whose therapeutic potential was demonstrated through network pharmacology analysis, reduces the levels of inflammatory cytokines (177). Other compounds such as thalidomide and aspirin, known for their roles in slowing down innate immunity, reducing inflammatory cytokines, and suppressing NF-κB activation, may be effective in treating rosacea (178, 179).

## 5 Conclusion/discussion

The diagnosis of rosacea primarily relies on patients’ symptoms and phenotypes, as outlined by the 2018 National Rosacea Society Expert Committee guidelines (46). Given the multifactorial nature of rosacea pathophysiology and the diverse triggers and mechanisms involved, treatment approaches must be personalized, taking into account predominant symptoms, phenotypes, pathogenic mechanisms, and trigger factors. Factors such as drug efficacy, tolerability, and previous treatment history should also be considered.

In addressing inflammatory lesions such as papules and pustules, treatment selection should align with underlying pathogenic mechanisms such as immune dysregulation or the presence of *Demodex* mites (46, 60, 180). Recommended topical treatments include ivermectin, azelaic acid, metronidazole, or microencapsulated benzoyl peroxide cream (69, 146, 157, 181). For severe cases, FDA-approved minocycline foam 1.5% or systemic doxycycline may be appropriate (46, 50). A network meta-analysis showed that, compared to low-dose doxycycline 40 mg, topical ivermectin, and metronidazole 0.75%, oral minocycline 100 mg had the best effect on papulopustules with a low incidence of side effects (81). There are also many off-label treatment options; for refractory papulopustules, oral isotretinoin is an option (60, 182). Oral macrolides can serve as secondary agents, particularly for patients with contraindications to tetracyclines, such as those who are refractory or pregnant (79). However, azithromycin has a high risk of adverse effects (RR, 8.9, 95% CI: 1.3-83), such as gastrointestinal issues (81). Topical pimecrolimus is also mentioned in the Swiss guidelines as an anti-inflammatory agent, but care must be taken due to its immunosuppressive effect, which may cause Demodex overgrowth and pose a potential risk of rosacea-like eruptions (91). Several treatments are still under investigation. As we gain a better understanding of rosacea’s pathophysiological pathways and develop new precision medicines, targets like the pro-inflammatory cytokine IL-17, activation of NF-κB, and the JAK-STAT signaling pathway are becoming therapeutic goals. Drugs such as secukinumab, novel proteasome inhibitors, and Janus kinase inhibitors are aimed at these targets and have shown promising results in novel research (86, 87, 97).

For persistent erythema or flushing, these symptoms are highly associated with neurovascular dysregulation (3). Therefore, drug mechanisms focus on promoting vasoconstriction and inhibiting various vasoactive neuropeptides. FDA-approved medications include brimonidine and oxymetazoline (46). Off-label use of propranolol and carvedilol has also been shown to help control persistent erythema (120). Additionally, many new drugs targeting neurogenic inflammation are under investigation, such as paroxetine, sumatriptan, monoclonal antibodies against CGRP, and botulinum toxin (28, 109, 110, 135). These medications aim to reduce neuropeptide-induced vascular dysfunction. However, the side effects and cost-effectiveness of these new drugs need to be considered, and their efficacy still requires validation through more extensive clinical trials.

Many therapeutic approaches coming down the pipeline address the central role of the immune and neurovascular systems in rosacea. Controlling the immune and neurovascular dysregulations will likely lead to increased improvement in rosacea signs and symptoms, and hopefully, with less side effects. Although some of the cited studies were conducted using animal models or at the cellular level, these references were included to illustrate the breadth of ongoing research and the potential for promising new treatment options for rosacea. However, these findings need validation and rigorous evidence to support their efficacy in humans. Future large-scale clinical trials will be essential to provide such evidence and confirm the effectiveness of these treatments in clinical practice. Understanding the triggers and concomitant medications may assist the clinician in selecting the appropriate treatment regimen for particular patients to maximize chances or rosacea symptom control. Research into correlation between genotypes and phenotypes in rosacea patients may also one day lead to a personalized approach to manage rosacea.

## Author contributions

K-YT: Visualization, Writing – original draft, Writing – review & editing. C-JJ: Writing – review & editing. Y-HS: Conceptualization, Writing – review & editing. AC: Conceptualization, Writing – review & editing.
