# Supplementary material for: Therapeutic strategies focusing on immune dysregulation and neuroinflammation in rosacea
Source: Front Immunol. 2024 Jul 29;15:1403798. doi: 10.3389/fimmu.2024.1403798 (PMC11317294; doi:10.3389/fimmu.2024.1403798)
Supplement: Supplementary file 1 [file Table_1.docx]

| **Supplementary Table 1 Oxford Centre for Evidence-Based Medicine 2011 Levels of Evidence (1)** | |
| --- | --- |
| Question | Does this intervention help? (Treatment benefits) |
| Level 1 | Systematic review of randomized trials or n-of-1 trials |
| Level 2 | Randomized trial or observational study with dramatic effect |
| Level 3 | Non-randomized controlled cohort/follow-up study |
| Level 4 | Case-series, case-control studies, or historically controlled studies |
| Level 5 | Mechanism-based reasoning |
| Reference: 1. Group OLoEW. " The Oxford 2011 Levels of Evidence." Oxford Centre for Evidence-Based Medicine. [*http://www*](http://www) *cebm net/index aspx? o= 5653* (2011). | |
