# Supplementary material for: Therapeutic strategies focusing on immune dysregulation and neuroinflammation in rosacea
Source: Front Immunol. 2024 Jul 29;15:1403798. doi: 10.3389/fimmu.2024.1403798 (PMC11317294; doi:10.3389/fimmu.2024.1403798)
Supplement: Supplementary file 2 [file Table_2.docx]

| **Supplementary Table 2 Selected therapeutics targeting immune dysregulation for rosacea. LOE= level of evidence, according to The Oxford 2011 Levels of Evidence (1)** | | | | | |
| --- | --- | --- | --- | --- | --- |
| **Agent** | **Rosacea population** | **Mechanism** | **Efficacy** | **Adverse event** | **Article** |
| **Systemic treatment** | | | | | |
| Minocycline extended-release oral capsule (DFD-29) | PPR | Inhibiting the innate immunity response, reducing the production of nitric oxide and reactive oxygen species, and suppression of the arachidonic acid pathway | DFD-29 40mg demonstrated significantly greater efficacy in improvement of IGA grade and reduction of lesion count than placebo | Gastrointestinal distress. Vestibular adverse effect like headache (4%) and vertigo (1.5%) (2). Dose-related phototoxicity. Rare events like lupus-like erythematosus, hyperpigmentation, and hepatotoxicity. | Tsianakas et al., 2021(2), phase I and II, LOE: 2 |
| Sarecycline | Moderate-severe PPR | Anti-inflammatory effect as tetracyclines | Significantly improves IGA grade, inflammatory lesion count, and rosacea secondary phenotypes like burning sensation | Gastrointestinal distress like nausea. Lower rate of adverse events associated with oral tetracyclines, such as photosensitivity, vertigo, and vaginal yeast infections. | Rosso et al., 2021(3), phase II, LOE:3 |
| Hydroxychloroquine 200 mg twice daily | ETR and PPR | Suppresses mast cells activation via inhibiting Ca2+ activated K + channel. Suppress the release of inflammatory factors, chemotaxis, degranulation and calcium influx. | IGA and CEA score showed a tendency to relief of rosacea (4). In terms of Rosacea-Specific Quality-of-Life scores, hydroxychloroquine showed noninferiority compared to the doxycycline group (5). | Gastrointestinal distress. Potential [ocular toxicity](https://www.sciencedirect.com/topics/pharmacology-toxicology-and-pharmaceutical-science/eye-toxicity) in long-term use | Li et al., 2020(6), clinical and experimental study, LOE: 3. Wang et al., 2019(7), pilot study, LOE: 3. |
| Secukinumab | Moderate to severe PPR | Inhibiting IL-17A and subsequent related cytokines, chemokines, and antimicrobial proteins | Global severity score and median Rosacea-Specific Quality-of-Life scores were significantly improved after 16 weeks of secukinumab | Most common adverse events were infections (39%). Other events like fatigue, rash (eczema) and pruritus. | Kumar et al., 2020(8), Exploratory study, LOE: 3. |
| Tofacitinib and abrocitinib | ETR, PPR and steroid-induced rosacea | Inhibiting JAK-STAT signaling pathway which plays a pivotal role in the proinflammatory processes within immune cells | 71.4% of 21 patients experiencing significant regression of facial erythema (IGA ≤ 1) and a mean change of -2.24 in the IGA score | Upper respiratory tract infection, diarrhea, hypertension and headache. Long-term use may cause malignancy, lymphopenia, gastrointestinal perforation. | Sun et al., 2022(9), case series, LOE:4. Xu et al., 2023(10), case series, LOE:4 |
| **Local treatment** | | | | | |
| Minocycline 1.5% foam (FMX) | Moderate to severe PPR | Minocycline's anti-inflammatory properties result from its ability to inhibit matrix metalloproteinases, immune cell function and infiltration, reduce oxidative stress, and inhibit nitric oxide synthase | Compared to control group, significantly reducing lesion number and improving symptoms without serious adverse effect. | Cutaneous adverse events such as pruritis, telangiectasia, dermatitis | Gold et al., 2020(11), phase III, LOE:2 |
| Minocycline gel 1% and 3% | PPR | Suppressing the production of pro-inflammatory cytokines(12), ROS(13), NOS(4), and MMPs(5) | Both concentrations of minocycline gel significantly reduced inflammatory lesion counts, with the 3% minocycline group showing a higher success rate in achieving the improvement of IGA grade at week 12 | Low rate of adverse events (3-5%) such as nausea, dermatitis, erythema, pruitus, hypersensitivity, headache, urticaria. | Webster et al., 2020(14), phase II, LOE:2 |
| Pimecrolimus and tacrolimus | PPR | Inhibiting T-cell activation, suppressing the synthesis of inflammatory cytokines, and preventing the release of cytokines from T cells and mast cells through calcineurin phosphatase inhibition | Pimicrolimus showed efficacy on significant improvement in erythema score and total rosacea severity score  Tacrolimus significantly improved erythema but not the number of papulopustular lesion | May cause burning, stinging, dryness, and itching sensation. Long-term use may cause rosacea-like eruption. | Karabulut et al., 2008(15), phase II, LOE:2 Bamford et al., 2004(16), open-label study, LOE:3 |
| Rapamycin | N/A | Hinders the mTORC1 signaling pathway, which controls cathelicidin expression in keratinocytes through a positive feedback loop involving TLR2. Diminishes LL37 levels and further reduces both NF-κB activation and the production of disease-specific cytokines and chemokines. | CEA and IGA scores were both significantly decreased | Most common adverse events were skin burning, pruritus, erythema, flu0like symptoms and skin infection. | Deng et al., 2021(17), clinical and experimental study, LOE:3 |
| Artemether emulsion (1%) | PPR | Anti-inflammatory effect and anti-Demodex folliculorum activity | Early improvement as early as 4 weeks, particularly in the papules and pustules scores. | Mild itching was present (4.8%). No edema, burning, or stinging, were reported through 12 weeks | Wang et al., 2019(18), phase II RCT, LOE: 2. |
| ACU-D1 | Moderate-severe PPR | Inhibiting the 26S proteasome results in NF-kB inhibition | Reducing inflammatory lesions and erythema in individuals with rosacea | 11% of skin related adverse events, including post dose facial perspiration and facial warmth | Jackson et al., 2021(19), phase I RCT, LOE: 2 |
| 5% dapsone gel | PPR | Anti-inflammatory effects are achieved by inhibiting the production of ROS, mitigating the impact of eosinophil peroxidase on mast cells, and suppressing inflammatory responses mediated by neutrophils | Comparable efficacy to 0.75% metronidazole gel in improving IGA scores and reducing the number of lesions | Totally 8.5% mild adverse events were observed including itching and burning | Faghih et al, 2015(20), phase II RCT, LOE:2 |
| ε-aminocaproic acid | PPR | Inhibit trypsin-like proteases in the stratum corneum, including KLK5 | IGA and CEA scores of subjects in the treatment group significantly improved at week 12 compared to baseline | N/A | Two et al., 2014(21), phase II, LOE:3 |
| Tranexamic acid | ETR | Repressing the angiogenesis by reducing the number of CD31^+^ cell and downregulating the expression levels of VEGF. Inhibiting the production of TLR2, pro-inflammatory cytokines (IL-6 and TNFα) | Significantly decreasing IGA score, redness score or redness area | Erythema, flushing, irritation, and a feeling of stinging/burning | Bageorgou et al., 2018(22), open label trial, LOE:3. Li et al., 2019(23), clinical and experimental study, LOE:3 |
| Abbreviation: LOE, level of evidence; PPR, Papulopustular rosacea; ETR, Erythematotelangiectatic rosacea; IGA, Investigator Global Assessment; ROS, reactive oxygen species; NOS, Nitric oxide species; MMP, matrix metalloproteinase; CEA, clinician severity assessment; IL-, interleukin-; JAK-STAT, Janus protein tyrosine kinase/Signal Transducers and Activators of Transcription; mTORC; TLR2, toll-like receptor 2; NF-κB, nuclear factor kappa-light-chain-enhancer of activated B cells; RCT, randomized controlled trial; KLK5, Kallikrein 5; TNF, tumor necrosis factor | | | | | |

Reference

1. Group OLoEW. " The Oxford 2011 Levels of Evidence." Oxford Centre for Evidence-Based Medicine. [*http://www*](about:blank) *cebm net/index aspx? o= 5653* (2011).

2. Tsianakas A, Pieber T, Baldwin H, Feichtner F, Alikunju S, Gautam A, et al. Minocycline Extended-Release Comparison with Doxycycline for the Treatment of Rosacea: A Randomized, Head-to-Head, Clinical Trial. *J Clin Aesthet Dermatol* (2021) 14(12):16-23. Epub 2022/02/01.

3. Rosso JQ, Draelos ZD, Effron C, Kircik LH. Oral Sarecycline for Treatment of Papulopustular Rosacea: Results of a Pilot Study of Effectiveness and Safety. *J Drugs Dermatol* (2021) 20(4):426-31. Epub 2021/04/15. doi: 10.36849/jdd.2021.5923.

4. Trachtman H, Futterweit S, Greenwald R, Moak S, Singhal P, Franki N, et al. Chemically Modified Tetracyclines Inhibit Inducible Nitric Oxide Synthase Expression and Nitric Oxide Production in Cultured Rat Mesangial Cells. *Biochem Biophys Res Commun* (1996) 229(1):243-8. Epub 1996/12/04. doi: 10.1006/bbrc.1996.1787.

5. Monk E, Shalita A, Siegel DM. Clinical Applications of Non-Antimicrobial Tetracyclines in Dermatology. *Pharmacol Res* (2011) 63(2):130-45. Epub 2010/10/13. doi: 10.1016/j.phrs.2010.10.007.

6. Li J, Yuan X, Tang Y, Wang B, Deng Z, Huang Y, et al. Hydroxychloroquine Is a Novel Therapeutic Approach for Rosacea. *Int Immunopharmacol* (2020) 79:106178. Epub 2020/01/10. doi: 10.1016/j.intimp.2019.106178.

7. Wang B, Yuan X, Huang X, Tang Y, Zhao Z, Yang B, et al. Efficacy and Safety of Hydroxychloroquine for Treatment of Patients with Rosacea: A Multicenter, Randomized, Double-Blind, Double-Dummy, Pilot Study. *J Am Acad Dermatol* (2021) 84(2):543-5. Epub 2020/05/23. doi: 10.1016/j.jaad.2020.05.050.

8. Kumar AM, Chiou AS, Shih YH, Li S, Chang ALS. An Exploratory, Open-Label, Investigator-Initiated Study of Interleukin-17 Blockade in Patients with Moderate-to-Severe Papulopustular Rosacea. *Br J Dermatol* (2020) 183(5):942-3. Epub 2020/05/05. doi: 10.1111/bjd.19172.

9. Sun YH, Man XY, Xuan XY, Huang CZ, Shen Y, Lao LM. Tofacitinib for the Treatment of Erythematotelangiectatic and Papulopustular Rosacea: A Retrospective Case Series. *Dermatol Ther* (2022) 35(11):e15848. Epub 2022/09/30. doi: 10.1111/dth.15848.

10. Xu B, Xu Z, Ye S, Sun H, Zhao B, Wu N, et al. Jak1 Inhibitor Abrocitinib for the Treatment of Steroid-Induced Rosacea: Case Series. *Front Med (Lausanne)* (2023) 10:1239869. Epub 2023/09/19. doi: 10.3389/fmed.2023.1239869.

11. Gold LS, Del Rosso JQ, Kircik L, Bhatia ND, Hooper D, Nahm WK, et al. Minocycline 1.5% Foam for the Topical Treatment of Moderate to Severe Papulopustular Rosacea: Results of 2 Phase 3, Randomized, Clinical Trials. *J Am Acad Dermatol* (2020) 82(5):1166-73. Epub 2020/02/01. doi: 10.1016/j.jaad.2020.01.043.

12. Cazalis J, Tanabe S, Gagnon G, Sorsa T, Grenier D. Tetracyclines and Chemically Modified Tetracycline-3 (Cmt-3) Modulate Cytokine Secretion by Lipopolysaccharide-Stimulated Whole Blood. *Inflammation* (2009) 32(2):130-7. Epub 2009/02/25. doi: 10.1007/s10753-009-9111-9.

13. Miyachi Y, Yoshioka A, Imamura S, Niwa Y. Effect of Antibiotics on the Generation of Reactive Oxygen Species. *J Invest Dermatol* (1986) 86(4):449-53. Epub 1986/04/01. doi: 10.1111/1523-1747.ep12285793.

14. Webster G, Draelos ZD, Graber E, Lee MS, Dhawan S, Salman M, et al. A Multicentre, Randomized, Double-Masked, Parallel Group, Vehicle-Controlled Phase Iib Study to Evaluate the Safety and Efficacy of 1% and 3% Topical Minocycline Gel in Patients with Papulopustular Rosacea. *Br J Dermatol* (2020) 183(3):471-9. Epub 2020/01/08. doi: 10.1111/bjd.18857.

15. Karabulut AA, Izol Serel B, Eksioglu HM. A Randomized, Single-Blind, Placebo-Controlled, Split-Face Study with Pimecrolimus Cream 1% for Papulopustular Rosacea. *J Eur Acad Dermatol Venereol* (2008) 22(6):729-34. Epub 2008/03/11. doi: 10.1111/j.1468-3083.2008.02589.x.

16. Bamford JT, Elliott BA, Haller IV. Tacrolimus Effect on Rosacea. *J Am Acad Dermatol* (2004) 50(1):107-8. Epub 2003/12/31. doi: 10.1016/s0190-9622(03)02157-1.

17. Deng Z, Chen M, Liu Y, Xu S, Ouyang Y, Shi W, et al. A Positive Feedback Loop between Mtorc1 and Cathelicidin Promotes Skin Inflammation in Rosacea. *EMBO Mol Med* (2021) 13(5):e13560. Epub 2021/03/19. doi: 10.15252/emmm.202013560.

18. Wang GJ, Gao XY, Wu Y, He HQ, Yu Y, Qin HH, et al. Evaluation of the Efficacy and Tolerance of Artemether Emulsion for the Treatment of Papulopustular Rosacea: A Randomized Pilot Study. *J Dermatolog Treat* (2019) 30(8):809-12. Epub 2019/04/25. doi: 10.1080/09546634.2019.1610549.

19. Jackson JM, Coulon R, Arbiser JL. Evaluation of a First-in-Class Proteasome Inhibitor in Patients with Moderate to Severe Rosacea. *J Drugs Dermatol* (2021) 20(6):660-4. Epub 2021/06/03. doi: 10.36849/jdd.2021.5925.

20. Faghihi G, Khosravani P, Nilforoushzadeh MA, Hosseini SM, Assaf F, Zeinali N, et al. Dapsone Gel in the Treatment of Papulopustular Rosacea: A Double-Blind Randomized Clinical Trial. *J Drugs Dermatol* (2015) 14(6):602-6. Epub 2015/06/20.

21. Two AM, Hata TR, Nakatsuji T, Coda AB, Kotol PF, Wu W, et al. Reduction in Serine Protease Activity Correlates with Improved Rosacea Severity in a Small, Randomized Pilot Study of a Topical Serine Protease Inhibitor. *J Invest Dermatol* (2014) 134(4):1143-5. Epub 2013/11/12. doi: 10.1038/jid.2013.472.

22. Bageorgou F, Vasalou V, Tzanetakou V, Kontochristopoulos G. The New Therapeutic Choice of Tranexamic Acid Solution in Treatment of Erythematotelangiectatic Rosacea. *J Cosmet Dermatol* (2019) 18(2):563-7. Epub 2018/08/14. doi: 10.1111/jocd.12724.

23. Li Y, Xie H, Deng Z, Wang B, Tang Y, Zhao Z, et al. Tranexamic Acid Ameliorates Rosacea Symptoms through Regulating Immune Response and Angiogenesis. *Int Immunopharmacol* (2019) 67:326-34. Epub 2018/12/24. doi: 10.1016/j.intimp.2018.12.031.
