# Supplementary material for: Therapeutic strategies focusing on immune dysregulation and neuroinflammation in rosacea
Source: Front Immunol. 2024 Jul 29;15:1403798. doi: 10.3389/fimmu.2024.1403798 (PMC11317294; doi:10.3389/fimmu.2024.1403798)
Supplement: Supplementary file 3 [file Table_3.docx]

| **Supplementary Table 3 Selected therapeutics targeting neurovascular dysregulation for rosacea. LOE= level of evidence, according to The Oxford 2011 Levels of Evidence (1)** | | | | | |
| --- | --- | --- | --- | --- | --- |
| **Agent** | **Rosacea patient population** | **Mechanism** | **Efficacy** | **Adverse event** | **Article** |
| **Systemic treatment** | | | | | |
| Paroxetine | Rosacea with refractory erythema | addressing dysfunction in 5-HT uptake, which can lead to abnormal dilation and constriction of blood vessels | Patients showed a significant improvement in erythema, flushing, burning sensation, and depression compared to the placebo group. | Most common adverse events were dizziness (10.3%), lethargy (10.3%), nausea (8.6%), dyspepsia (6.9%), and muscle tremors (5.2%) | Wang et al., 2023(2), phase II, LOE:2 |
| Sumatriptan | Erythematotelangiectatic rosacea | Sumatriptan may inhibit PACAP38- mediated rosacea exacerbations either via action on mast cells or possibly via direct inhibition of neuropeptides | Compared with placebo, sumatriptan significantly reduced PACAP38-induced facial skin blood flow, reduced duration of flushing and facial edema. | Injection site reactions, tingling, dizziness, and triptan sensations (including tingling, feeling heaviness, or pressure) | Wienholtz et al., 2021(3), phase I, clinical experimental study, LOE: 2 |
| CGRP monoclonal antibody | Patients with migraine and rosacea | Inhibiting CGRP which belongs to vasoactive neurotransmitter, leading to vasodilation and a cascade of inflammatory reactions in rosacea skin lesion | significant improvement in severity scores compared to before treatment | Upper respiratory tract infection, nausea, constipation, fatigue, and the possibility of development of hypertension are the associated side effects | Sia et al., 2023(4), case series, LOE:4 |
| **Local treatment** | | | | | |
| Botulinum toxin | Erythematotelangiectatic rosacea | blocking the release of ACh, CGRP, VIP, substance P, and glutamate, or by reducing non-noxious stimulation | In comparison to the control group, the experimental group exhibited heightened hydration levels, along with reductions in the global flushing symptom score, VISIA red value, erythema index, transepidermal water loss, and sebum secretion. | The most common side effect was localized pain, while the rarer and more concerning side effect was paralysis of motor muscles (4.3%)(5) | Tong et al., 2022(6), phase 2, LOE:3 |
| Abbreviation: 5-HT, 5-hydroxytryptamine; LOE, level of evidence; PACAP, Pituitary adenylate-cyclase-activating polypeptide; CGRP, Calcitonin gene-related peptide; Ach, acetylcholine; VIP, vasoactive intestinal polypeptide; | | | | | |

References

1. Group OLoEW. " The Oxford 2011 Levels of Evidence." Oxford Centre for Evidence-Based Medicine. [*http://www*](http://www) *cebm net/index aspx? o= 5653* (2011).

2. Wang B, Huang Y, Tang Y, Zhao Z, Shi W, Jian D, et al. Paroxetine Is an Effective Treatment for Refractory Erythema of Rosacea: Primary Results from the Prospective Rosacea Refractory Erythema Randomized Clinical Trial. *J Am Acad Dermatol* (2023) 88(6):1300-7. Epub 2023/02/23. doi: 10.1016/j.jaad.2023.01.044.

3. Wienholtz NKF, Christensen CE, Coskun H, Zhang DG, Ghanizada H, Egeberg A, et al. Infusion of Pituitary Adenylate Cyclase-Activating Polypeptide-38 in Patients with Rosacea Induces Flushing and Facial Edema That Can Be Attenuated by Sumatriptan. *J Invest Dermatol* (2021) 141(7):1687-98. Epub 2021/02/19. doi: 10.1016/j.jid.2021.02.002.

4. Sia T, Webb T, Li S, Moskatel LS, Chang ALS. An Exploratory Comparative Case Series of Calcitonin Gene-Related Peptide Monoclonal Antibodies in Patients with Migraine with Rosacea. *Br J Dermatol* (2023) 189(6):776-8. Epub 2023/08/19 20:42. doi: 10.1093/bjd/ljad277.

5. Alsaati AA, Alsaadoun D, Kinkar LI, Alkhamis RS, Ahmed WA, Almathami AH. The Efficacy and Safety of Botulinum Toxin a for the Treatment of Rosacea: A Systematic Review. *Cureus* (2023) 15(12):e51304. Epub 2024/01/30. doi: 10.7759/cureus.51304.

6. Tong Y, Luo W, Gao Y, Liu L, Tang Q, Wa Q. A Randomized, Controlled, Split-Face Study of Botulinum Toxin and Broadband Light for the Treatment of Erythematotelangiectatic Rosacea. *Dermatol Ther* (2022) 35(5):e15395. Epub 2022/02/22. doi: 10.1111/dth.15395.
