# Supplementary material for: Therapeutic strategies focusing on immune dysregulation and neuroinflammation in rosacea
Source: Front Immunol. 2024 Jul 29;15:1403798. doi: 10.3389/fimmu.2024.1403798 (PMC11317294; doi:10.3389/fimmu.2024.1403798)
Supplement: Supplementary file 4 [file Table_4.docx]

| **Supplementary Table 4 Selected therapeutics targeting microbiota for rosacea. LOE= level of evidence, according to The Oxford 2011 Levels of Evidence (1)** | | | | | |
| --- | --- | --- | --- | --- | --- |
| **Agent** | **Rosacea patient population** | **Mechanism** | **Efficacy** | **Adverse event** | **Article** |
| **Topical therapy** | | | | | |
| Benzoyl Peroxide Cream, 5% | moderate to severe rosacea | Oxidizing agent with anti-bacterial properties | IGA scores and inflammatory lesion counts significantly decreased after 12 weeks of treatment in comparison with the vehicle-controlled arm. | Erythema (2.3%), pain (1.6%), pruritus (1.2%), and edema | Bhatia et al., 2023(2), phase II, LOE:2 |
| Omiganan gel | severe papulopustular rosacea | The mechanism of action involves rapid bactericidal and fungicidal effects against a wide range of infectious organisms | Reducing mean inflammatory lesion counts and achieving a higher proportion of improvement in IGA score at week 12 compared to baseline | N/A | Grada et al., 2019(3), phase III, LOE:2 |
| Rifaximin | N/A | Eradicating SIBO, which might disrupt immunity, triggering rosacea by increasing tumor necrosis factor-alpha or other cytokines, suppressing interleukin-17, and stimulating the T helper 1-mediated immune response | Significant reduction in cutaneous lesions with the use of Rifaximin (1200 mg/day) for 10 days when compared to the control group | Headache, peripheral edema, nausea and dizziness | Parodi et al., 2008(4), pilot study, LOE:3 |
| Abbreviation: IGA, Investigator Global Assessment; LOE, level of evidence; SIBO, Small intestinal bacterial overgrowth | | | | | |

References

1. Group OLoEW. " The Oxford 2011 Levels of Evidence." Oxford Centre for Evidence-Based Medicine. [*http://www*](http://www) *cebm net/index aspx? o= 5653* (2011).

2. Bhatia ND, Werschler WP, Baldwin H, Sugarman J, Green LJ, Levy-Hacham O, et al. Efficacy and Safety of Microencapsulated Benzoyl Peroxide Cream, 5%, in Rosacea: Results from Two Phase Iii, Randomized, Vehicle-Controlled Trials. *J Clin Aesthet Dermatol* (2023) 16(8):34-40. Epub 2023/08/28.

3. Grada A, Van Doorn M, Lain E, Furst K, Feiss G. Lb1092 Topical Omiganan for Severe Papulopustular Rosacea: A Randomized, Vehicle-Controlled, Double-Blind, Multicenter Study. *Journal of Investigative Dermatology* (2019) 139(9):B13.

4. Parodi A, Paolino S, Greco A, Drago F, Mansi C, Rebora A, et al. Small Intestinal Bacterial Overgrowth in Rosacea: Clinical Effectiveness of Its Eradication. *Clin Gastroenterol Hepatol* (2008) 6(7):759-64. Epub 2008/05/06. doi: 10.1016/j.cgh.2008.02.054.
