# Supplementary material for: Therapeutic strategies focusing on immune dysregulation and neuroinflammation in rosacea
Source: Front Immunol. 2024 Jul 29;15:1403798. doi: 10.3389/fimmu.2024.1403798 (PMC11317294; doi:10.3389/fimmu.2024.1403798)
Supplement: Supplementary file 5 [file Table_5.docx]

| **Supplementary Table 5 Selected promising therapeutics for rosacea in pre-clinical study** | | | | | |
| --- | --- | --- | --- | --- | --- |
| **Agent** | **Route** | **Sample characteristics** | **Mechanism** | **Efficacy** | **Article** |
| **Pre-clinical study** | | | | | |
| Osthole | IP | C57BL/6 and Balb/c mice and Human LAD2 mast cells | Reducing both the initial (Ca2+ release and degranulation) and later phases (production of cytokines) of mast cell activation through MRGPRX2 in an in vitro setting | Suppressing inflammation in mouse models of pseudo-allergy that depends on MrgpB2 | Callahan  et al., 2020(1) |
| Celastrol | IP | LL37-induced rosacea-like mice | -Inhibiting the LL37-activated Ca^2+^/CaMKII-mTOR-NF-κB pathway  -Suppressing the expression of inflammatory cytokines, Th17 immune response and cutaneous angiogenesis | Celastrol ameliorated erythema, skin thickness and inflammatory cell infiltration in the dermis of LL37-treated mice | Zeng et al., 2022(2) |
| Coptis chinensis Franch | N/A | human epidermal keratinocytes | -Downregulating the expression of KLK5, cathelicidin, and TLR2.  -Inhibiting of LL-37 processing and the proliferation of human microvascular endothelial cells | Improved rosacea by regulating the immune response and angiogenesis | Roh et al., 2020(3) |
| Paeoniflorin | N/A | RAW 264.7 (2 × 10^5^) mouse monocyte line | Promoting SOCS3 expression in RAW 264.7 cells and inhibiting the increase in TLR-2 and LL37 expression through the ASK1-p38 cascade, thereby alleviating the macrophage-related inflammatory response | Inhibiting the macrophage-related rosacea-like inflammatory response through the SOCS3-ASK1-p38 pathway | Liu et al., 2021(4) |
| Erythroid differentiation regulator 1 | Intra-dermal | BALB/c mice with LL-37 peptide induction | rErdr1 suppressed CD4+ and CD8+ T-cell infiltration and VEGF expression | Resulting in a significant reduction of erythema, inflammatory cell infiltration, and microvessel density | Kim et al., 2015(5) |
| Aryl hydrocarbon Receptor agonist, benvitimod | Topical | BALB/c mice with LL-37 peptide induction | The increased levels of TLR2 and chemokines (CCL5, CXCL9, CXCL10, and CXCL11) induced by LL-37 treatment were reduced upon AhR activation | Improving rosacea-like eruptions induced by LL-37 in mice, as indicated by reductions in redness scores, redness areas, and dermal inflammatory cell infiltrates | Sun et al., 2022(6) |
| Metformin | N/A | BALB/c mice with LL-37 peptide induction/ HaCaT cells and HUVEC cells | Metformin suppressed CD4+ T cell, VEGF expression, LL37- and TNF-α-induced the ROS production and MAPK-NF-κB signal activation | Metformin treatment significantly improved rosacea-like skin issues, reducing immune cell infiltration, cytokine/chemokine expression, and angiogenesis | Li et al., 2021(7) |
| Pioglitazone nanoemulsion | Topical | BALB/c mice | Significantly reducing the levels of cytokines IL-6, IL-1β, and TNF-α. | Sligh reduction of redness, and notable improving inflammation | Espinoza  Et al., 2019(8) |
| Melatonin | N/A | N/A | -Repressing the expression of IL-6, TNF-α, TLR2, TGF-β1, MMP9, and VEGF in rosacea-like dermatitis  -Reducing the infiltration of CD4^+^ T cells | The redness area, redness score, skin thickness, and inflammatory cell infiltration were dramatically decreased by melatonin treatment | Zhang et al., 2021(9) |
| Thalidomide | IP | LL37-induced rosacea-like mice | -Decreasing the production of cytokines and chemokines induced by LL37  -Reducing CD4^+^ T helper cell infiltration and downregulating Th1- and Th17-polarizing genes  -Lowering the microvessel density and VEGF expression  -Suppressing NF-κB activation | Significantly alleviating erythema and reducing inflammatory cell infiltration in dermis | Chen et al., 2019(10) |
| Aspirin | IP | LL37-induced rosacea-like mice | -Suppressing the production of chemokines and cytokines associated with rosacea, and the Th1- and Th17-polarized immune responses  -Decreasing the microvessels density and the VEGF expression  -Inhibiting the activation of NF-κB signaling | Improvement in erythema and telangiectasia, redness area, and score occurs in a dose-dependent manner. Histopathologically, a reduction in immune cell infiltration can also be observed. | Deng et al., 2021(11) |
| Abbreviation: IP, intre-peritoneal; MRGPRX2, Mas-related G-protein coupled receptor member X2; mTOR, mammalian target of rapamycin, NF-κB, nuclear factor kappa-light-chain-enhancer of activated B cells ; KLK-5, Kallikrein 5; TLR2, toll-like receptor; N/A; non-applicable; SOCS3, suppressor of cytokine signaling 3; ASK1, Apoptosis Signal-regulating Kinase 1; CCL, CC chemokine ligand; CXCL, C-X-C motif ligand; AhR, aryl hydrocarbon receptor; ROS, reactive oxygen species; MAPK, Mitogen-activated protein kinases; IL-interleukin; TNF, tumor necrosis factor; VEGF, vascular endothelial growth factor; TGF, Transforming growth factor; MMP, matrix metalloproteinase; Th-, T helper | | | | | |

References:

1. Callahan BN, Kammala AK, Syed M, Yang C, Occhiuto CJ, Nellutla R, et al. Osthole, a Natural Plant Derivative Inhibits Mrgprx2 Induced Mast Cell Responses. *Front Immunol* (2020) 11:703. Epub 2020/05/12. doi: 10.3389/fimmu.2020.00703.

2. Zeng Q, Yang J, Yan G, Zhang L, Wang P, Zhang H, et al. Celastrol Inhibits Ll37-Induced Rosacea by Inhibiting Ca(2+)/Camkii-Mtor-Nf-Κb Activation. *Biomed Pharmacother* (2022) 153:113292. Epub 2022/06/20. doi: 10.1016/j.biopha.2022.113292.

3. Roh KB, Ryu DH, Cho E, Weon JB, Park D, Kweon DH, et al. Coptis Chinensis Franch Directly Inhibits Proteolytic Activation of Kallikrein 5 and Cathelicidin Associated with Rosacea in Epidermal Keratinocytes. *Molecules* (2020) 25(23). Epub 2020/12/02. doi: 10.3390/molecules25235556.

4. Liu Z, Zhang J, Jiang P, Yin Z, Liu Y, Liu Y, et al. Paeoniflorin Inhibits the Macrophage-Related Rosacea-Like Inflammatory Reaction through the Suppressor of Cytokine Signaling 3-Apoptosis Signal-Regulating Kinase 1-P38 Pathway. *Medicine (Baltimore)* (2021) 100(3):e23986. Epub 2021/02/07. doi: 10.1097/md.0000000000023986.

5. Kim M, Kim KE, Jung HY, Jo H, Jeong SW, Lee J, et al. Recombinant Erythroid Differentiation Regulator 1 Inhibits Both Inflammation and Angiogenesis in a Mouse Model of Rosacea. *Exp Dermatol* (2015) 24(9):680-5. Epub 2015/05/06. doi: 10.1111/exd.12745.

6. Sun Y, Chen L, Wang H, Zhu P, Jiang S, Qi R, et al. Activation of Aryl Hydrocarbon Receptor Ameliorates Rosacea-Like Eruptions in Mice and Suppresses the Tlr Signaling Pathway in Ll-37-Induced Hacat Cells. *Toxicol Appl Pharmacol* (2022) 451:116189. Epub 2022/08/05. doi: 10.1016/j.taap.2022.116189.

7. Li Y, Yang L, Wang Y, Deng Z, Xu S, Xie H, et al. Exploring Metformin as a Candidate Drug for Rosacea through Network Pharmacology and Experimental Validation. *Pharmacol Res* (2021) 174:105971. Epub 2021/11/12. doi: 10.1016/j.phrs.2021.105971.

8. Espinoza LC, Silva-Abreu M, Calpena AC, Rodríguez-Lagunas MJ, Fábrega MJ, Garduño-Ramírez ML, et al. Nanoemulsion Strategy of Pioglitazone for the Treatment of Skin Inflammatory Diseases. *Nanomedicine* (2019) 19:115-25. Epub 2019/04/21. doi: 10.1016/j.nano.2019.03.017.

9. Zhang H, Zhang Y, Li Y, Wang Y, Yan S, Xu S, et al. Bioinformatics and Network Pharmacology Identify the Therapeutic Role and Potential Mechanism of Melatonin in Ad and Rosacea. *Front Immunol* (2021) 12:756550. Epub 2021/12/14. doi: 10.3389/fimmu.2021.756550.

10. Chen M, Xie H, Chen Z, Xu S, Wang B, Peng Q, et al. Thalidomide Ameliorates Rosacea-Like Skin Inflammation and Suppresses Nf-Κb Activation in Keratinocytes. *Biomed Pharmacother* (2019) 116:109011. Epub 2019/05/28. doi: 10.1016/j.biopha.2019.109011.

11. Deng Z, Xu S, Peng Q, Sha K, Xiao W, Liu T, et al. Aspirin Alleviates Skin Inflammation and Angiogenesis in Rosacea. *Int Immunopharmacol* (2021) 95:107558. Epub 2021/03/21. doi: 10.1016/j.intimp.2021.107558.
